# The potential impact of COVID-19 and diabetes on intervertebral disc degeneration

**DOI:** 10.31744/einstein_journal/2021CE6911

**Published:** 2021-12-14

**Authors:** Luciano Rodrigo Lopes, Silvana Kertzer Kasinski

**Affiliations:** 1 Universidade Federal de São Paulo São Paulo SP Brazil Universidade Federal de São Paulo, São Paulo, SP, Brazil.

Dear Editor,

A large part of the world's population has become infected by severe acute respiratory syndrome coronavirus 2 (SARS-CoV-2), the causative agent of coronavirus disease 2019 (COVID-19).^( 1 )^ While most SARS-CoV-2 infected individuals were asymptomatic or presented with mild symptoms, some were severely affected. The most common COVID-19 symptoms include fever, cough, pneumonia, dyspnea and acute lung injury. Although respiratory symptoms are more frequent, extrapulmonary conditions including coagulation disorders, cardiac injury, kidney failure, and metabolic disorder, can also occur in severe COVID-19. Moreover, the SARS-CoV-2 infection induces an immune system overreaction, with high levels of inflammatory cytokines, chemokines, and free radicals, causing severe injuries to the lungs and other organs.^( 1 , 2 )^ The uncontrolled production of pro-inflammatory cytokines induced by SARS-CoV-2 is called a cytokine storm, a hyperimmune state in patients with severe disease.

*Diabetes mellitus* (DM), one of the main comorbidities associated with COVID-19, contributes to immune dysregulation and exacerbates inflammatory reactions during SARS-CoV-2 infection. Coronavirus disease 2019 associated with DM enhances metabolic disruption and increases glycemia, which impairs the clinical course of the disease.^( 2 , 3 )^ The dysregulated glucose metabolism and increased pro-inflammatory cytokines in patients with DM and COVID-19 accelerate free radicals production.^( 2 )^ Oxidative stress damages proteins, lipids, and DNA, and affects the structure and function of various organs and tissues.^( 2 )^ SARS-CoV-2 directly affects the lungs, myocardial muscles, kidneys, liver, and other tissues. However, the effect of SARS-CoV-2 infection on intervertebral discs (IVD) remains unknown.

Some pathophysiological effects during the clinical course of severe COVID-19 potentially contribute to IVD degeneration. The etiology of IVD degeneration is characterized by high levels of pro-inflammatory cytokines and oxidative stress mediators.^( 4 )^ Moreover, DM significantly contributes to IVD degenerative disease. The high sugars levels in DM can glycate proteins or lipids, which are called advanced glycation end-products (AGEs). Increased IVD degeneration occurs due to AGEs accumulation, triggering inflammatory reactions through pro-inflammatory cytokines and free radicals.^( 5 )^

**Figure 1:**
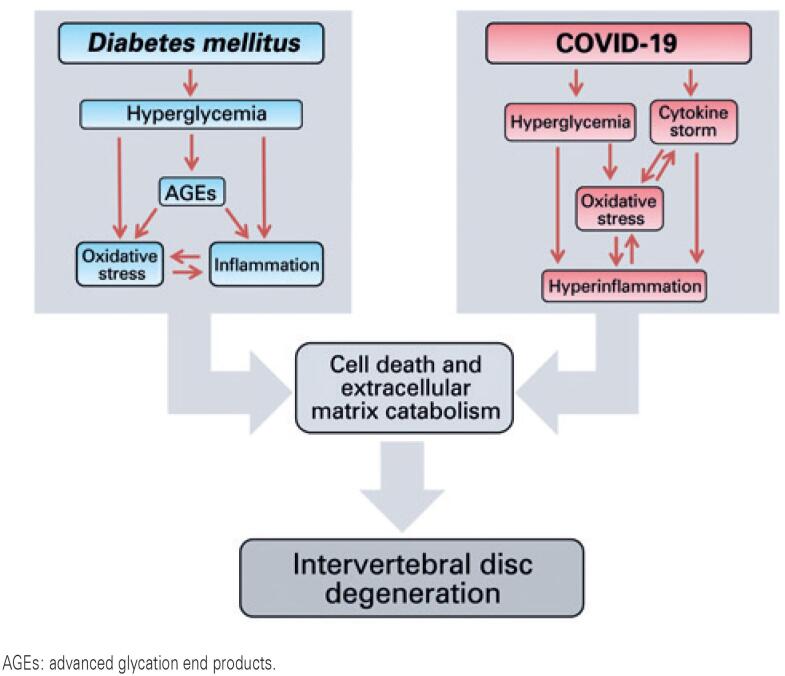
*Diabetes mellitus* and COVID-19 pathways may lead to intervertebral discs degeneration

The combination of COVID-19 and DM promotes inflammatory reactions and oxidative stress, which drive IVD degeneration ( Figure 1 ). Subsequently, these processes can cause cell death and lead IVD to a catabolic state, reducing the extracellular matrix contributing to its degeneration. Thus, COVID-19 must be considered, in addition to DM, as an essential factor that promotes IVD degeneration. Considering the large number of COVID-19 patients and the long duration of severe SARS-CoV-2 infection, IVD degenerative disease should be considered a potential sequela in cured patients. Hence, effective interventions to attenuate the effect of COVID-19 on IVD should be established, since IVD degenerative disease causes suffering and distress to patients and their families.
