# Supplementary material for: The potential impact of COVID-19 and diabetes on intervertebral disc degeneration
Source: Einstein (Sao Paulo). 2021 Dec 14;19:eCE6911. doi: 10.31744/einstein_journal/2021CE6911 (PMC8687644; doi:10.31744/einstein_journal/2021CE6911)
Supplement: Translation

# Translation: Impacto potencial da COVID-19 e do diabetes na degeneração do disco intervertebral

**Authors:** Luciano Rodrigo Lopes, Silvana Kertzer Kasinski

**Affiliations:** 1 São Paulo SP Brasil Universidade Federal de São Paulo, São Paulo, SP, Brasil.

Prezado Editor,

Grande parte da população mundial foi infectada pelo coronavírus da síndrome respiratória aguda grave 2 (SARS-CoV-2), agente causador da doença pelo coronavírus 2019 (COVID-19).^( 1 )^Embora a maioria dos indivíduos infectados pelo SARS-CoV-2 tivesse sido assintomática ou apresentasse sintomas leves, alguns deles foram gravemente afetados. Os sintomas mais comuns da COVID-19 incluem febre, tosse, pneumonia, dispneia e lesão pulmonar aguda. Embora os sintomas respiratórios sejam os mais frequentes, alterações extrapulmonares, incluindo distúrbios de coagulação, lesão cardíaca, insuficiência renal e distúrbios metabólicos, também podem ocorrer na COVID-19 grave. Além disso, a infecção por SARS-CoV-2 induz a uma reação exagerada do sistema imunológico, com altos níveis de citocinas inflamatórias, quimiocinas e radicais livres, que causam lesões graves nos pulmões e em outros órgãos.^( 1 , 2 )^A produção descontrolada de citocinas pró-inflamatórias induzida pelo SARS-CoV-2 é chamada de tempestade de citocinas, um estado hiperimune em pacientes com doença grave.

O *diabetes mellitus* (DM), uma das principais comorbidades associadas à COVID-19, contribui para a desregulação imunológica e exacerba as reações inflamatórias durante a infecção pelo SARS-CoV-2. A COVID-19 associada ao DM piora os distúrbios metabólicos e aumenta a glicemia, prejudicando o curso clínico da doença.^( 2 , 3 )^O metabolismo desregulado da glicose e o aumento das citocinas pró-inflamatórias em pacientes com DM e COVID-19 aceleram a produção de radicais livres.^( 2 )^O estresse oxidativo causa dano a proteínas, lipídios e DNA, além de afetar a estrutura e a função de vários órgãos e tecidos.^( 2 )^O SARS-CoV-2 afeta diretamente os pulmões, os músculos do miocárdio, os rins, o fígado e outros tecidos. No entanto, o efeito da infecção pelo SARS-CoV-2 nos discos intervertebrais (DIV) permanece desconhecido.

Alguns efeitos fisiopatológicos durante o curso clínico da COVID-19 grave contribuem potencialmente para a degeneração do DIV. A etiologia da degeneração do DIV é caracterizada por altos níveis de citocinas pró-inflamatórias e mediadores de estresse oxidativo.^( 4 )^Além disso, o DM contribui significativamente para a doença degenerativa do DIV. Os altos níveis de açúcar no DM podem glicar proteínas ou lipídios, que são chamados de produtos finais da glicação avançada (AGEs - *advanced glycation end-products* ). O aumento da degeneração do DIV ocorre devido ao acúmulo de AGEs, desencadeando reações inflamatórias por meio de citocinas pró-inflamatórias e radicais livres.^( 5 )^

A combinação de COVID-19 e DM promove reações inflamatórias e estresse oxidativo, que levam à degeneração do DIV ( Figura 1 ). Posteriormente, esses processos podem causar morte celular e levar o DIV a um estado catabólico, reduzindo a matriz extracelular e contribuindo para sua degeneração. Assim, a COVID-19 deve ser considerada, além do DM, um fator essencial que promove a degeneração do DIV. Considerando-se o grande número de pacientes com COVID-19 e a longa duração da infecção grave por SARS-CoV-2, a doença degenerativa do DIV deve ser considerada uma sequela em potencial nos pacientes curados. Portanto, devem ser estabelecidas intervenções efetivas para atenuar o efeito da COVID-19 no DIV, uma vez que a doença degenerativa do DIV causa sofrimento e angústia para os pacientes e suas famílias.

**Figura 1:** Vias do *diabetes mellitus* e COVID-19 podem levar à degeneração dos discos intervertebrais
